# Association between geriatric nutritional risk index and depression prevalence in the elderly population in NHANES

**DOI:** 10.1186/s12889-024-17925-z

**Published:** 2024-02-14

**Authors:** Zijiao Li, Li Zhang, Qiankun Yang, Xiang Zhou, Meng Yang, Yu Zhang, Youzan Li

**Affiliations:** 1 Nephrology department of the First Affiliated Hospital of Army Medical University, 400038 Chongqing, China; 2 Department of Neurosurgery, National Clinical Research Center for Child Health and Disorders, Ministry of Education Key Laboratory of Child Development and Disorders, Chongqing Key Laboratory of Pediatrics, Children’s Hospital of Chongqing Medical University, 400014 Chongqing, China; 3 Department of Dermatology, The Second Affiliated Hospital of Chongqing Medical University, 400010 Chongqing, China; 4 National & Regional United Engineering Lab of Tissue Engineering, Department of Orthopedics, Southwest Hospital, Army Medical University, 400038 Chongqing, China

**Keywords:** Geriatric nutritional risk index, Depression, Older adults, NHANES database, Depression prevention, Statistical analysis

## Abstract

**Background:**

The prevalence of depression is increasing in the elderly population, and growing evidence suggests that malnutrition impacts mental health. Despites, research on the factors that predict depression is limited.

**Methods:**

We included 2946 elderly individuals from National Health and Nutrition Examination Survey (NHANES) spanning the years 2011 through 2014. Depressive symptoms were assessed using the PHQ-9 scale. Multinomial logistic regression was performed to evaluate the independent association between Geriatric Nutritional Risk Index (GNRI) and depression prevalence and scores. Subgroup analysis was conducted to explore potential factors influencing the negative correlation between GNRI and depression. Restricted cubic spline graph was employed to examine the presence of a non-linear relationship between GNRI and depression.

**Results:**

The depression group had a significantly lower GNRI than the non-depression group, and multivariate logistic regression showed that GNRI was a significant predictor of depression (*P* < 0.001). Subgroup analysis revealed that certain demographic characteristics were associated with a lower incidence of depression in individuals affected by GNRIs. These characteristics included being female (*P* < 0.0001), non-Hispanic black (*P* = 0.0003), having a moderate BMI (*P* = 0.0005), having a college or associates (AA) degree (*P* = 0.0003), being married (*P* = 0.0001), having a PIR between 1.50 and 3.49 (*P* = 0.0002), being a former smoker (*P* = 0.0002), and having no history of cardiovascular disease (*P* < 0.0001), hypertension (*P* < 0.0001), and diabetes (*P* = 0.0027). Additionally, a non-linear negative correlation (non-linear *P* < 0.01) was found between GNRI and depression prevalence, with a threshold identified at GNRI = 104.17814.

**Conclusion:**

The GNRI demonstrates efficacy as a reliable indicator for forecasting depression in the elderly population. It exhibits a negative nonlinear correlation with the prevalence of depression among geriatric individuals.

## Introduction

Depression is a prevalent mental health disorder that is widespread in various regions of the world. Among the elderly populace, depression is also highly prevalent, with a global prevalence rate of 28.4% from 2000 to 2021 [1]. Due to the lack of specific diagnostic criteria for depression in older adults, elderly individuals with depression are underdiagnosed and do not receive timely treatment, leading to significant public health issues that affect individuals, healthcare systems, and society as a whole [2]. Studies have indicated that depression is a recurrent and chronic disease associated with increased risks of death, metabolic diseases, cardiovascular diseases, and cancer mortality [3]. The increasing prevalence of depression calls for the need for timely, preventive methods or indicators. Some studies have reported potentially protective effects of dietary quality on depression symptoms, as well as the biological actions of nutrients [4]. It is crucial to understand the intervention of dietary quality on the risk of depression during adulthood [5].

The influence of diet on depression has received considerable attention in the past five years. In addition to single nutrients, the effect of the entire dietary pattern on depression has been of particular interest [6]. However, research on the role of nutrition in depression is scarce. The Geriatric Nutritional Risk Index (GNRI), as initially documented in 2005, functions as a tool for evaluating nutritional status among geriatric individuals [7]. Multiple studies have found that GNRI is closely related to the prognosis of many diseases, such as diabetes, heart failure, cancer, and osteoporosis [8]. In addition to the strong prognostic value in different disease populations, GNRI has gradually gained attention due to its ease of use in clinical analysis and its objective calculation based on available data [9].

The National Health and Nutrition Examination Survey (NHANES) (https://www.cdc.gov/nchs/nhanes/index.htm) is a comprehensive cross-sectional survey that collects information on the health and nutrition of the United States household population. The NHANES database has extensive sample coverage and various indicators, providing access to population statistics, socioeconomic, dietary, and health, physiological measurements, laboratory tests, and other information from all over the United States. In this study, employing the NHANES large-scale cross-sectional study, we identified the correlation between GNRI and depression scores and depression prevalence in elderly people, which can serve as a predictive tool for depression.

## Methods

### Database and survey populations

**Fig. 1:**
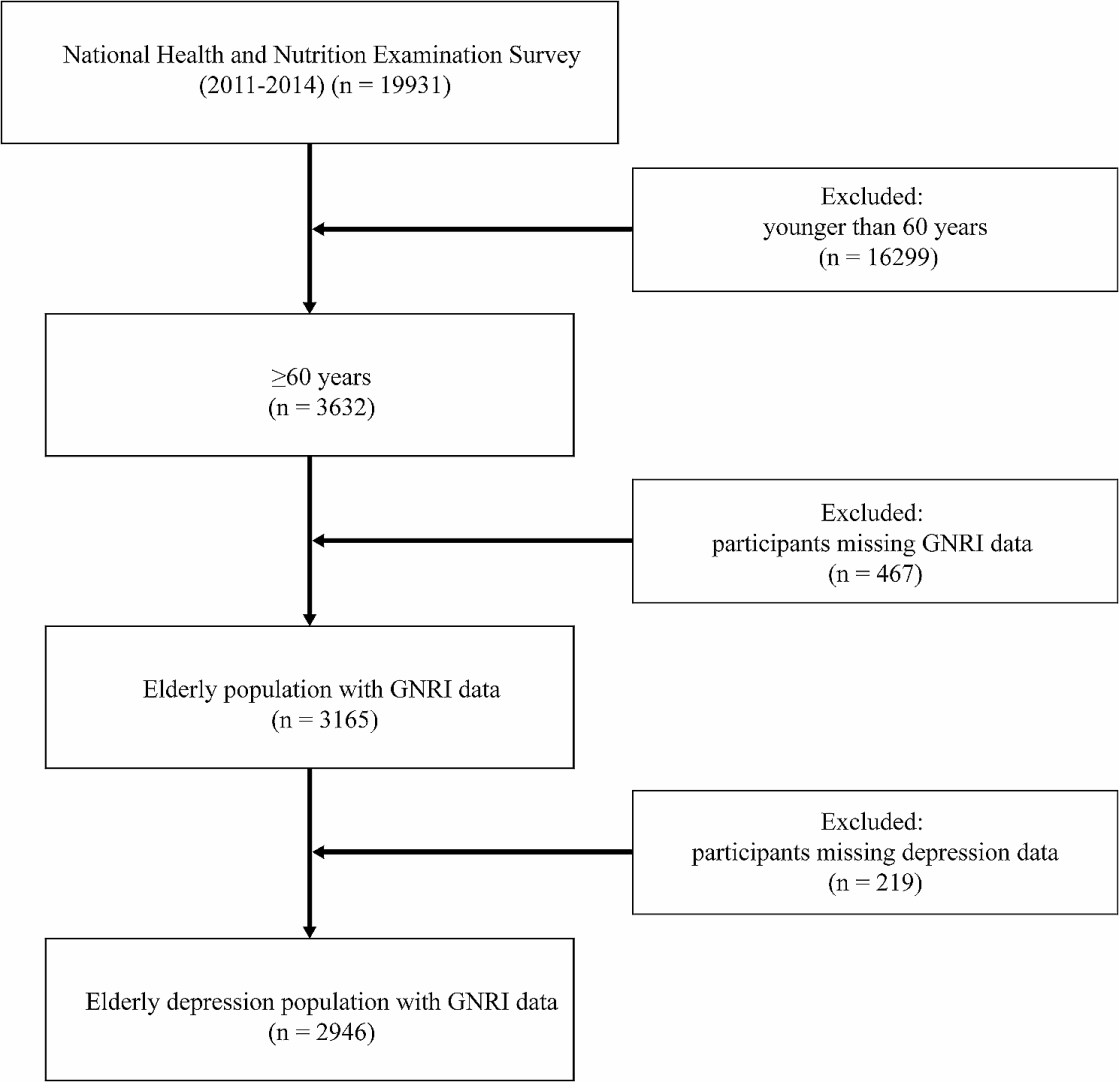
Participant recruitment standard flow chart

The official NHANES website (https://www.cdc.gov/nchs/nhanes/index.htm) provided the information used in this research. The National Health and Nutrition Examination Study (NHANES), a thorough study carried out by the Centers for Disease Control and Prevention (CDCP) and the National Center for Health Statistics (NCHS), seeks to provide nationally representative statistics on the civilian population of the United States. All participants provided informed permission before their interviews and tests, and the NCHS Ethics Review Board approved the data-collecting procedure for the NHANES. In this investigation, a dataset was created by extracting responses from the NHANES publicly accessible data files from 2011 to 2014. A total of 19,931 individuals participated in two consecutive NHANES survey cycles. This study excluded 16,299 individuals who were under the age of 60. Subsequently, individuals who did not have calculated GNRI-related data (*n* = 467) and those who were missing data on depressive symptoms (*n* = 219) were also excluded. The final study population consisted of 2,946 individuals. Figure 1 illustrates a flowchart that describes the criteria used for patient selection.

### GNRI evaluation and grouping

The GNRI was calculated based on several factors, including the subject’s height (m), weight (kg), optimum weight (kg), and serum albumin (g/L) [7, 10]. The following is the GNRI calculating formula: GNRI = 1.489 × Albumin (g/L) + 41.7 × [body weight/ideal body weight], ideal body mass = 22 × Height (m) × Height (m). The weight/ideal weight ratio is set to 1 when the actual weight is greater than the desired weight. The patients included in this study were divided into two groups: High-GNRI group (GNRI ≥ 98) and Low-GNRI group (GNRI < 98) [9].

### Definition of depression

Depressive symptoms were assessed using the 9-item Patient Health Questionnaire (PHQ-9) Depression Scale, which consists of 9 questions based on the Diagnostic and Statistical Manual of Mental Disorders, Fourth Edition (DSM-IV) Depressive Symptoms [11]. Respondents were asked to rate the severity of their symptoms from 0 (not at all) to 3 (almost every day). The total score for the nine items ranged from 0 to 27 [12]. In this study, individuals with a total score greater than 9 on the PHQ-9 were considered to have clinically relevant depressive disorder (CRD), and a total score of 9 or less was considered non-clinical [13]. In the study conducted by Kurt Kroenke et al., it was found that using the mental health professional (MHP) reinterview as the criterion standard, a PHQ-9 score ≥ 10 demonstrated a sensitivity of 88% and a specificity of 88% for major depression [14].

### Covariates

Interviewers ascertained a participant’s birthdate and other demographic data by self-report through the initial screening questionnaire. Age in years was a continuous variable. Gender was reported as male or female. Race was classified as Mexican American, other Hispanic, non-Hispanic white, non-Hispanic black, or other. Body mass index (BMI) was calculated by the following formula: BMI = weight (kg)/height2 (m2). The Systemic Immune-Inflammation Index (SII) level was determined by multiplying the platelet count by the neutrophil count/lymphocyte count [4]. Education attainment was assessed through the initial screening questionnaire and categorized as less than 9th grade, 9th-11th grade, high school, some college or associate degree, college or above. Marital status was categorized as married, widowed, divorced, separated, never married, or living with a partner. Household income is classified based on the poverty income ratio (PIR) into three categories: PIR < 1.5,1.5 ≤ PIR < 3.5, or PIR ≥ 3.5. Smoking status was established by responses to: “Do you now smoke cigarettes?” and “Do you now smoke cigarettes?”. The classification of one’s smoking status was based on the duration and number of cigarettes they have consumed throughout their lifetime. There are three distinct categories of individuals who engage in smoking: “never,” “former,” and “current.” (never, smoked less than 100 cigarettes; former, smoked more than 100 cigarettes in life and smoke not at all now; now, smoked more than 100 cigarettes in life and smoke some days or every day) [15].

The study categorized participants based on their average daily alcohol consumption into three groups: ‘mild,’ ‘moderate,’ and ‘heavy.’ Those in the ‘moderate’ category consumed 2 drinks per day for women and 3 drinks per day for men, while individuals in the ‘heavy’ category consumed at least 3 drinks per day for women and at least 4 drinks per day for men. Participants who did not fall into either of these categories were considered mild drinkers. The study aimed to determine the occurrence of cardiovascular disease (CVD) outcomes by self-reported diagnoses of five major CVD events, which involved congestive heart failure (CHF), coronary heart disease (CHD), angina pectoris, heart attack, and stroke. Participants who answered yes to the question,' Have you ever been told by a physician that you had CHD/CHF/angina/a heart attack or a stroke?’ were identified as having CVD. It is noteworthy that relying on self-reported measures can introduce recall bias, which can affect data interpretation [16]. Hypertension is diagnosed based on four criteria, and individuals who meet any one of these criteria are categorized as hypertensive patients. The diagnostic criteria consist of self-reported hypertension history, the utilization of antihypertensive medication, a systolic blood pressure (SBP) ≥ 140mmHg, or a diastolic blood pressure (DBP) ≥ 90mmHg [17]. The diagnosis of hyperlipidemia refers to 6 criteria, and participants who meet any one of these criteria are defined as hyperlipidemia patients. The criteria for diagnosing hyperlipidemia are as follows: (1) total cholesterol ≥ 200 mg/dL, (2) triglycerides ≥ 150 mg/dL, (3) HDL cholesterol < 40 mg/dL for males and < 50 mg/dL for females, (4) LDL ≥ 130 mg/dL, (5) participants using lipid-lowering medications, or (6) patients self-reporting a diagnosis of hyperlipidemia [18]. Participants who meet the following two criteria are defined as diabetes patients. Diabetes diagnosis criteria were (1) participants using antidiabetic medications; or (2) patients self-reporting a diabetes diagnosis.

### Statistical analysis

The study employed the R software (version 4.2.3) developed by the R project (http://www.r-project.org) for data analysis. The specific R packages used in this study are as follows: “doBy” package, which provides functionality for grouping data frames by factor variables; “plotrix” package, used to create various charts; “stringi” and “stringr” packages, both used for manipulating and operating on strings; “rms” package, used for fitting predictive models; “car” package, used for testing various regression models; “kableExtra” package, used for creating and customizing aesthetically appealing and readable tables, and “readxl package, used for reading Microsoft Excel files in R (.xls and.xlsx formats). To address the complexities inherent in survey designs, survey non-response, and post-stratification adjustments, NHANES created weights using the Mobile Examination Center (MEC) sample weights and the relevant home-examined sample design variables (strata, primary sampling unit). These weights were utilized to ensure representative results, aligning with the demographic characteristics of the entire United States population. Consequently, all analyses presented in this study were conducted by appropriately weighting the samples in accordance with NHANES analytical guidelines [19].

Initially, the sample was divided into two groups based on the depression threshold: depressed and non-depressed. Continuous variables are expressed as weighted means ± standard deviations. *P* value was calculated by weighted linear regression model. Categorical variables were expressed as numbers and percentages and compared using the chi-square test. Multivariable logistic regression analysis was used to study the association between GNRI and depression. Furthermore, subgroup analysis and interaction tests were performed according to age, gender, race, education, marital status, BMI, smoking, hypertension, cardiovascular disease, hyperlipidemia, and diabetes. Ultimately, through the construction of a restricted cubic spline plot, we aim to ascertain whether there exists a non-linear correlation between the GNRI and depression prevalence.

## Result

### Baseline characteristics

Table 1 presents the weighted characteristics of participants divided into two groups based on their depression status. A total of 2946 participants took part in the study, out of which 280 individuals were identified as adults with suggested depression. In the primary analysis, age (69.453 ± 6.72 vs. 67.945 ± 6.59, *P* = 0.00136) emerged as a significant factor influencing depression. The average age of participants with depression was lower compared to those without depression, suggesting that individuals with depression were younger. Body mass index (BMI), a measure of obesity, was found to be significantly different between the two groups (28.834 ± 6.11 vs. 31.241 ± 7.69, *P* < 0.00001). The data showed that the average BMI of the depression group was higher than that of the non-depression group, indicating a tendency for higher obesity levels among depressed patients. Moreover, the depressed group displayed a higher proportion of individuals with low education levels, while the non-depressed group was primarily composed of individuals with a high school education or higher. This suggests that education level may be associated with the risk of depression (*P* < 0.00001). Additionally, the depressed group had a higher proportion of individuals who were divorced or widowed, while the non-depressed group had a higher proportion of individuals who were married (*P* < 0.00001). Changes in marital status may consequently influence mental health.

**Table 1:** Baseline characteristics of the study participants

Variables	Depression		***P*** value
	No	Yes	
**n**	2666	280	
**Age, years**	69.453 ± 6.72	67.945 ± 6.59	0.00136
**BMI, kg/m²**	28.834 ± 6.11	31.241 ± 7.69	< 0.00001
**GNRI**	104.343 ± 4.32	102.853 ± 5.20	< 0.00001
**SII**	565.649 ± 411.04	580.343 ± 432.70	0.61189
**Gender, n(%)**			0.00044
Female	1243(46.61%)	96(34.34%)	
male	1423(53.39%)	184(65.66%)	
**Race, n(%)**			0.00002
Mexican American	88(3.29%)	19(6.89%)	
Other Hispanic	92(3.45%)	22(7.84%)	
Non-Hispanic White	2129(79.86%)	187(66.83%)	
Non-Hispanic Black	211(7.92%)	34(12.32%)	
Other Race	146(5.48%)	17(6.12%)	
**Education, n(%)**			< 0.00001
Less than 9th grade	161(6.05%)	41(14.78%)	
9th-11th grade	266(9.97%)	50(17.97%)	
High school	584(21.89%)	63(22.44%)	
Some college or AA degree	829(31.1%)	93(33.21%)	
College graduate or above	825(30.96%)	32(11.59%)	
NA	1(0.02%)	0(%)	
**Marital Status, n(%)**			< 0.00001
Married	1684(63.17%)	123(44.06%)	
Widowed	446(16.74%)	74(26.29%)	
Divorced	323(12.1%)	51(18.23%)	
Separated	29(1.08%)	9(3.07%)	
Never married	115(4.32%)	16(5.84%)	
Living with partner	68(2.56%)	6(2.32%)	
NA	1(0.03%)	1(0.19%)	
**PIR, n(%)**			< 0.00001
< 1.50	529(19.83%)	132(47.03%)	
1.50–3.49	936(35.1%)	96(34.34%)	
≥ 3.50	1202(45.07%)	52(18.63%)	
**Smoke, n(%)**			0.03358
Never Smoke	1349(50.6%)	125(44.66%)	
Former Smoke	1028(38.57%)	109(39.07%)	
Current Smoke	289(10.84%)	46(16.27%)	
**Alcohol Use, n(%)**			0.05074
Never Alcohol	1951(73.18%)	197(70.32%)	
Former Alcohol	496(18.59%)	42(14.9%)	
Current Alcohol	219(8.23%)	41(14.79%)	
**CVD, n(%)**			< 0.00001
No	2112(79.22%)	181(64.5%)	
Yes	554(20.78%)	99(35.5%)	
**Hypertension, n(%)**			0.04001
No	902(33.83%)	76(27.04%)	
Yes	1764(66.17%)	204(72.96%)	
**Hyperlipidemia, n(%)**			0.64783
No	377(14.15%)	37(13.04%)	
Yes	2289(85.85%)	243(86.96%)	
**Diabetes, n(%)**			0.00038
No	2134(80.06%)	196(69.97%)	
Yes	532(19.94%)	84(30.03%)	

${\chi ^2}$ analysis is used to test significance between groups for categorical variables. GNRI: Geriatric Nutritional Risk Index. SII: Systemic Immune-Inflammation Index

BMI: Body Mass Index. PIR: Poverty-Income Ratio. CVD: Cardiovascular Disease

Regarding financial factors, the depressed group exhibited a higher proportion of individuals with a lower household income ratio (PIR), while the no-depression group displayed the opposite pattern. Financial difficulties may contribute to psychological stress and the onset of depression (*P* < 0.00001). Analysis of disease factors and unhealthy habits indicated that the number of smokers among individuals with depression was higher compared to those without depression (16.27% vs. 10.84%, *P* = 0.03358). Similarly, the prevalence of cardiovascular disease (CVD) among the depressed group was higher than the non-depressed group (35.5% vs. 20.78%, *P* < 0.00001). Additionally, the prevalence of hypertension was higher in the depressed group (72.96% vs. 66.17%, *P* = 0.04001), as well as the presence of diabetes (30.03% vs. 19.94%, *P* = 0.00038). These findings demonstrate significant differences in baseline characteristics among all variables except alcohol consumption and hyperlipidemia status (*P* < 0.05).

### Distribution of GNRI in non-depressed and depressed individuals

**Fig. 2:**
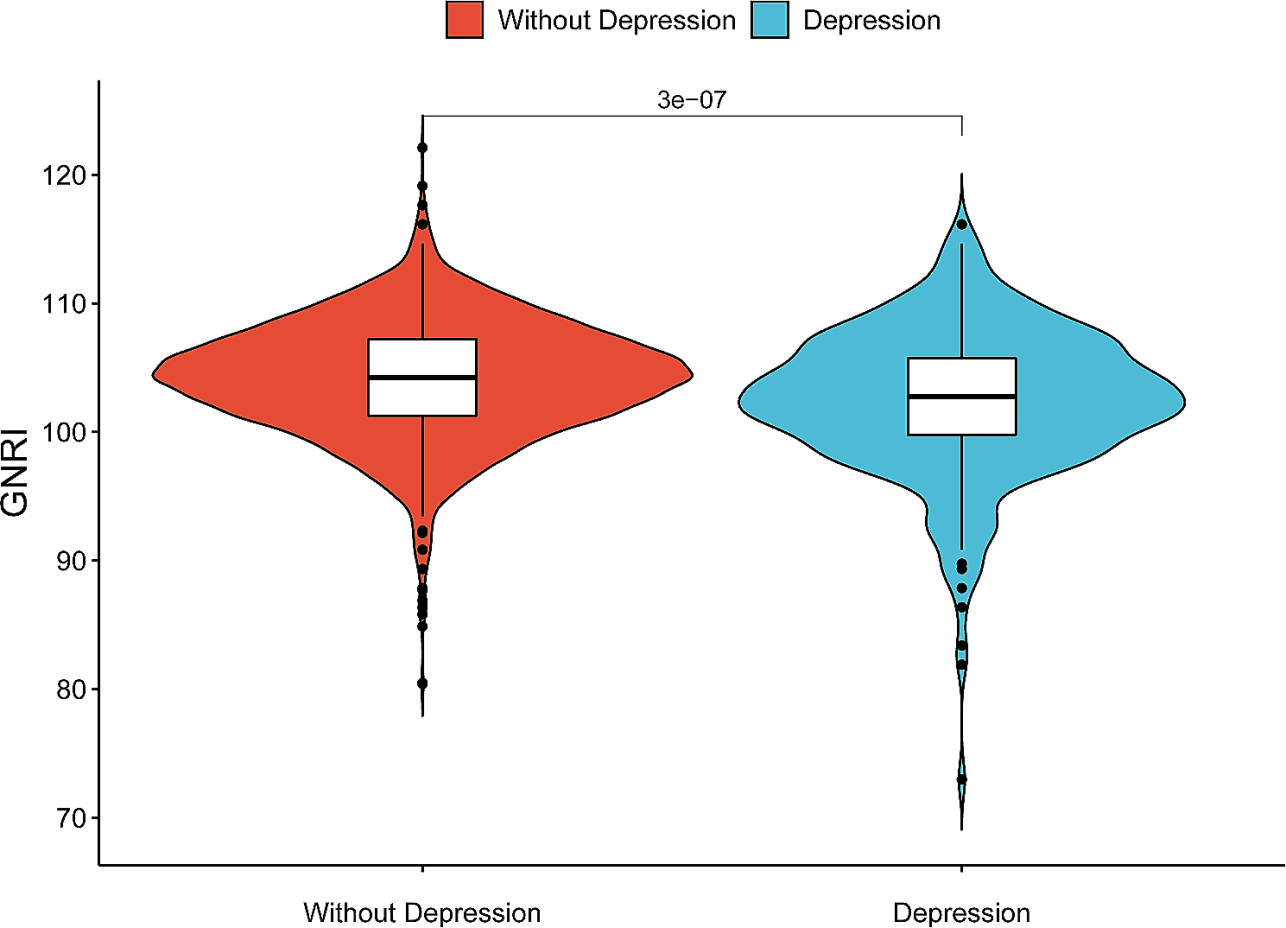
Violin plot of GNRI distribution in non-depressed and depressed subjects

The GNRI in non-depressed individuals exhibited a higher value compared to that in depressed individuals. This disparity was found to be statistically significant (*P* = 3 × 10^ (-7)), and the two groups showed an overall tendency towards tighter clustering with minimal dispersion (Fig. 2).

### The relationship between GNRI levels and depression

Multivariable logistic regression analysis (Table 2) was used to investigate the association between GNRI and depression scores (DPs). In the unadjusted model, the results showed a significant negative association between GNRI levels and DPs in the individual without depression (OR -0.062, *P* < 0.00001). However, no significant negative correlation between GNRI levels and DPs was observed in individuals with depression (OR = -0.021, *P* = 0.58193). There existed a significant negative correlation between GNRI levels and DPs in the overall sample (OR -0.056, *P* < 0.00001). In the adjusted model I, age, gender, and race were considered as adjustment variables. Adjusted model II involved additional adjustment for BMI, education, marital status, PIR, smoking, CVD, diabetes, and hypertension, in addition to age, sex, and race. The results demonstrated consistency with the unadjusted model. Consequently, a preliminary inference can be drawn that there exists an inverse association between GNRI level and depression scores, indicating that individuals with lower GNRI levels are more susceptible to depression and GNRI can act as predictive factors for depression. However, in depressed patients, GNRI cannot be utilized to predict the severity of depression due to the absence of a significant association between depression scores and GNRI.

**Table 2:** Logistic regression analysis for associations between GNRI and DP

Model	Without Depression		With Depression		Total	
	OR (95% CI)	***P***-value	OR (95% CI)	***P***-value	OR (95% CI)	***P***-value
Non-adjusted	-0.062 (-0.083, -0.042)	< 0.00001	-0.021 (-0.097, 0.054)	0.58193	-0.056 (-0.076, -0.036)	< 0.00001
Adjust I	-0.057 (-0.078, -0.036)	< 0.00001	-0.022 (-0.098, 0.055)	0.58012	-0.054 (-0.074, -0.033)	< 0.00001
Adjust II	-0.042 (-0.064, -0.021)	0.00014	-0.075 (-0.163, 0.013)	0.09478	-0.045 (-0.067, -0.024)	0.00003

Non-adjusted model adjusts for: None

Adjust I model adjust for: age; gender; race

Adjust II model adjust for: age; gender; race; BMI; education; marital status; poverty income ratio (PIR); smoke; cardiovascular disease (CVD); diabetes (DIQ); hypertension

Table 3 presents the results of a logistic regression analysis examining the relationship between the high nutritional risk index (High-GNRI) and the prevalence of depression, which is measured as a dichotomous variable. The variable adjustments of the three models given in the table are consistent with Table 2. The analysis of the data revealed varying degrees of negative correlations between high GNRI levels and depression in the three models, using the Low Nutritional Risk Index (Low GNRI) as a reference. The OR values and *P* values indicated that when GNRI increases by one unit, the risk of depression will be reduced by 0.447 times (*P* < 0.00001), 0.436 times (*P* < 0.00001), and 0.564 times (*P* = 0.00693) in the three models. In summary, a high nutritional risk index may serve as a protective factor against the development of depression.

**Table 3:** Logistic regression analysis for associations between High-GNRI and depression

Exposure	Non-adjusted		Adjust I		Adjust II	
	OR (95% CI)	***P***-value	OR (95% CI)	***P***-value	OR (95% CI)	***P***-value
Low GNRI	1		1		1	
High GNRI	0.447 (0.311, 0.643)	0.00001	0.436 (0.300, 0.633)	0.00001	0.564 (0.373, 0.855)	0.00693

Non-adjusted model adjusts for: None

Adjust I model adjust for: age; gender; race

Adjust II model adjust for: age; gender; race; BMI; education; marital status; poverty income ratio (PIR); smoke; cardiovascular disease (CVD); diabetes (DIQ); hypertension

Low GNRI was the control group

### Subgroup analysis

**Fig. 3:**
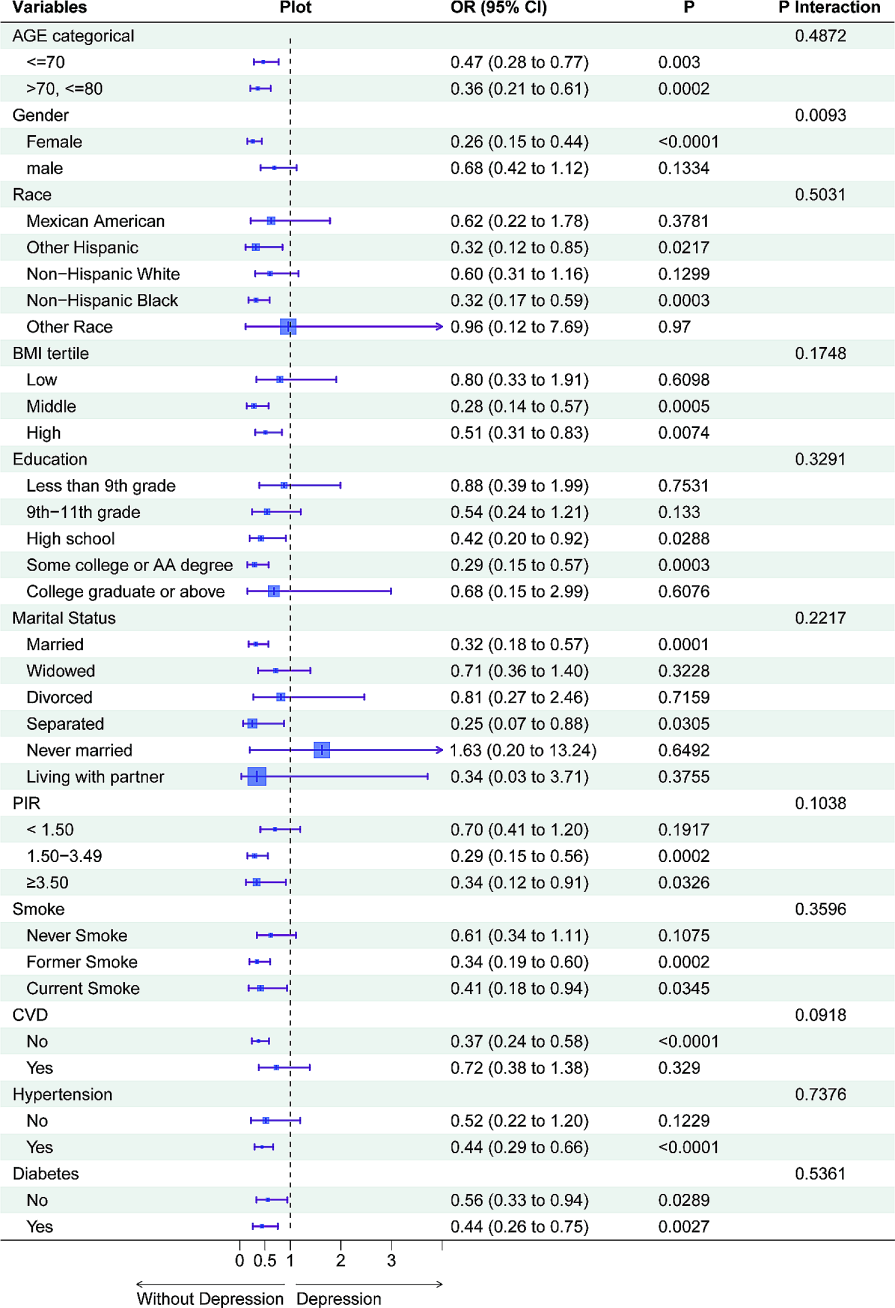
Forest plot of high GNRI subgroup analysis using low GNRI as reference

Through stratified analysis, each sample in the study was categorized and analyzed independently to ascertain the influence of confounding factors and specific population. The results presented in Table 4 (with Fig. 3 depicting the forest plot post data visualization) reveal that age has been segregated into two groups, ≤ 70 and > 70, ≤80, with corresponding OR and *P* values of (0.466 and 0.003) and (0.359 and 0.0002), respectively. Further, the data suggest that certain demographic groups, including women (*P* < 0.0001), non-Hispanic blacks (*P* = 0.0003), those with middle BMI (*P* = 0.0005), some college or AA degree (*P* = 0.0003), married (*P* = 0.0001), PIR between 1.50 and 3.49 (*P* = 0.0002), former smokers (*P* = 0.0002), those without CVD (*P* < 0.0001), those with hypertension (*P* < 0.0001) and diabetes (*P* = 0.0027), could be a significantly relevant population that is particularly vulnerable to the impact of GNRI, leading to a decreased occurrence of depression.

**Table 4:** Subgroup logistic regression analysis for the association between High-GNRI and depression

	OR (95% CI)	***P***-value	Interaction ***P***-value
**AGE categorical**			0.4872
<=70	0.466 (0.281, 0.772)	0.003	
> 70, <=80	0.359 (0.210, 0.613)	0.0002	
**Gender**			0.0093
Female	0.257 (0.150, 0.441)	< 0.0001	
male	0.685 (0.417, 1.123)	0.1334	
**Race**			0.5031
Mexican American	0.623 (0.218, 1.784)	0.3781	
Other Hispanic	0.319 (0.120, 0.846)	0.0217	
Non-Hispanic White	0.599 (0.309, 1.163)	0.1299	
Non-Hispanic Black	0.322 (0.175, 0.594)	0.0003	
Other Race	0.961 (0.120, 7.688)	0.97	
**BMI**			0.1748
Low	0.796 (0.332, 1.910)	0.6098	
Middle	0.281 (0.138, 0.573)	0.0005	
High	0.509 (0.310, 0.835)	0.0074	
**Education**			0.3291
Less than 9th grade	0.877 (0.386, 1.990)	0.7531	
9th-11th grade	0.542 (0.244, 1.205)	0.133	
High school	0.422 (0.195, 0.915)	0.0288	
Some college or AA degree	0.293 (0.150, 0.573)	0.0003	
College graduate or above	0.678 (0.154, 2.989)	0.6076	
**Marital Status**			0.2217
Married	0.319 (0.179, 0.568)	0.0001	
Widowed	0.713 (0.364, 1.395)	0.3228	
Divorced	0.814 (0.269, 2.463)	0.7159	
Separated	0.250 (0.071, 0.878)	0.0305	
Never married	1.627 (0.200, 13.244)	0.6492	
Living with partner	0.339 (0.031, 3.713)	0.3755	
**PIR**			0.1038
< 1.50	0.699 (0.408, 1.197)	0.1917	
1.50–3.49	0.294 (0.154, 0.559)	0.0002	
≥ 3.50	0.336 (0.124, 0.914)	0.0326	
**Smoke**			0.3596
Never Smoke	0.614 (0.340, 1.112)	0.1075	
Former Smoke	0.342 (0.194, 0.602)	0.0002	
Current Smoke	0.411 (0.180, 0.937)	0.0345	
**CVD**			0.0918
No	0.374 (0.241, 0.580)	< 0.0001	
Yes	0.725 (0.380, 1.383)	0.329	
**Hypertension**			0.7376
No	0.517 (0.224, 1.195)	0.1229	
Yes	0.442 (0.295, 0.662)	< 0.0001	
**Diabetes**			0.5361
No	0.559 (0.332, 0.942)	0.0289	
Yes	0.442 (0.259, 0.754)	0.0027	

Low-GNRI was the control group

### Nonlinear relationship between GNRI and depression prevalence in the elderly

**Fig. 4:**
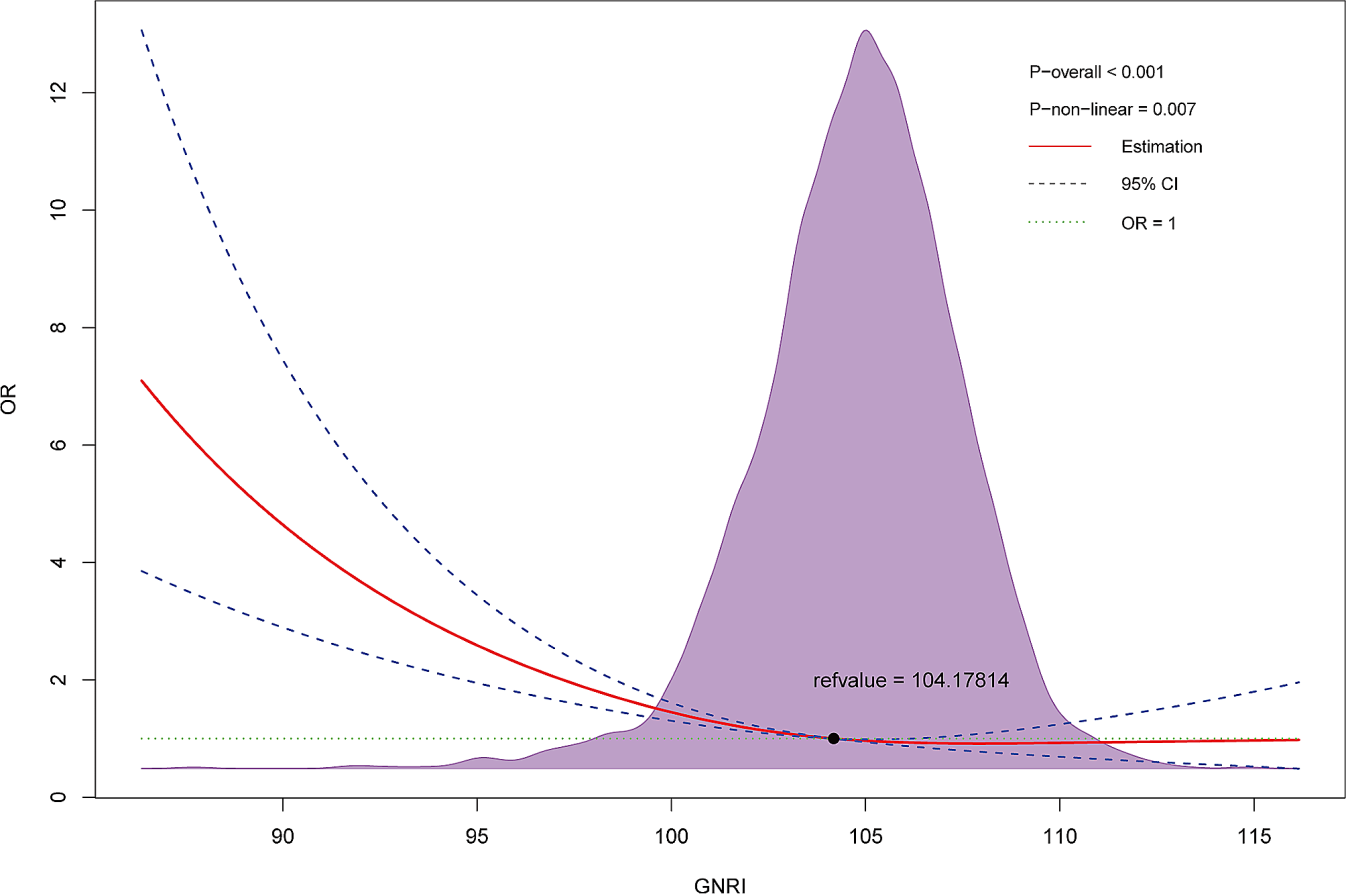
Restricted spline regression showed the correlation between GNRI and depression in the elderly

The restricted spline regression model has revealed a nonlinear, negative correlation between the GNRI and the prevalence of depression in the elderly population (nonlinear *P* = 0.013). Moreover, after controlling for confounding factors (age, sex, and race), GNRI was found to have an inverse association with the risk of depression (nonlinear *P* = 0.007; Fig. 4). As GNRI increases, the prevalence of depression gradually decreases, provided that GNRI is less than 104.17814.

## Discussion

Depression is a common mental health disorder with complex etiology and a significant impact on public health. Early identification of individuals at risk of depression is crucial for effective intervention and prevention [20]. As of January 2023, a total of 20 models predicting the onset of depression have been documented. Risk factors incorporated into these models comprise age, physical health, and cognitive function, among others. But there remains a paucity of research examining the relationship between dietary information and depression prediction [21]. GNRI is an indicator used to evaluate the nutritional and functional status of elderly individuals, integrates weight changes and serum albumin levels for this purpose. A study conducted on a sample of 307 patients who had suffered from ischemic stroke (with a mean age of 63 years) revealed that low serum pre-albumin levels at admission were linked to post-stroke depression reported 30 months following the onset of the stroke [9]. Previous studies with small sample sizes have demonstrated the potential of serum vitamin D and homocysteine levels in predicting the occurrence of post-stroke depression [22, 23]. However, the existing literature still lacks adequate clinical research and sufficient sample sizes to directly determine whether malnutrition can serve as a predictive factor or worsen the condition of depression.

This paper endeavors to present an analysis of data on the elderly population, featuring 2,946 cases, out of which 280 are depression patients, drawn from the NHANES database covering the period between 2011 and 2014. We employed the PHQ-9 sample table to evaluating depressive symptoms, quantify depression and its severity. Through logistic regression, we examined the interrelationship between GNRI and depression scores. We discovered that in the population without depression, there was a negative correlation between the GNRI and the depression score. This indicates that with a decrease in GNRI, depression score is likely to increase. Additionally, the significance levels (*P* values) of the three adjusted models were very small (*P* < 0.00001, *P* < 0.00001, *P* = 0.00014), demonstrating statistical significance. In the overall sample, a negative correlation between the GNRI and depression score also existed (*P* < 0.00001, *P* < 0.00001, *P* = 0.00003). However, there was no statistical significance in the depression population. The findings suggest that the GNRI can be a predictor for the presence of depression in elderly patients, with the risk of depression increasing as the GNRI index decreases. However, in the depressed patients, it cannot reflect the magnitude of depression based on quantified depression scores. Binary variable regression analysis results between high GNRI and depression indicate a negative correlation between high GNRI and depression, using low GNRI as the benchmark.

Malnutrition of the elderly can lead to weakened immunity, poor disease resistance, muscle atrophy, low body temperature, osteoporosis, cognitive impairment, increased risk of falls, and premature death. Some clinical studies have found a complex relationship between BMI and the development of depression, which may vary in different populations (Asian and European), and environments and other social factors also affect the relationship between BMI and depression [24]. There is no clear evidence in the literature regarding the relationship between educational level and depression. Our study suggests that people with higher education are less likely to suffer from depression. Marital status has a significant impact on depression. People aged 45 and above who are separated/divorced/widowed/unmarried may be at high risk of depression. Measures such as ensuring adequate sleep, relieving pain, and improving quality of life can alleviate depression symptoms in these individuals [25]. Our study results suggest that currently, married elderly people can lower their probability of developing depression by improving their GNRI. Regarding the impact of smoking on depression, most studies focus on secondhand smoke [26, 27]. Smoking has various states, including quitting, previously smoking, and repeated quitting, so more accurate data are required to describe the smoking situation.

The result provides some suggestions for policy and research directions. At the policy level, it is highly necessary to include nutritional assessment in the routine health check-ups for the elderly, which may require considering nutritional factors when updating guidelines and protocols. Strengthening health education for the elderly, especially emphasizing information related to nutrition and mental health, is meaningful. This can be achieved through conducting promotional activities in the community, medical institutions, and educational institutions to increase awareness among the elderly about the importance of nutrition, thereby preventing potential symptoms of depression. As for the research direction, the impact of nutrition on depression in the elderly population needs to be emphasized. Whether this phenomenon occurs in young people or other specific groups has not been determined. In addition, more long-term studies are needed to confirm the causal relationship between nutritional indices and depression. This study has some limitations. Firstly, it is a cross-sectional investigation which prevents us from determining the causal relationship between GNRI and depression. Specifically, it is uncertain whether a higher GNRI is a determinant of reduced prevalence of depression or if depression itself exacerbates malnutrition. Additionally, the data used in the study is based solely on the NHANES study and collected nutritional data and depression scores at a single time point, which may not fully reflect individuals’ long-term status and changes. The depression score is assessed only through some subjective questions, which may result in response bias or other potential influencing factors. To fully comprehend and apply the study’s findings, a multicenter longitudinal clinical trial would be necessary. Such trials could track changes in participants’ nutritional levels and depression symptoms and conduct long-term follow-ups. Such a trial would permit the tracking of participants’ nutritional levels and depression symptoms, as well as the conducting of long-term follow-ups. Employing this methodological design, it is possible to better appreciate the specific impact of nutritional status on the development and progression of depression in older adults and to more accurately gauge the relationship between GNRI and depression prevalence.

## Conclusion

This study utilized a cross-sectional national-level approach, using data from the NHANES database from 2011 to 2014. After applying selection criteria, the final sample included 2946 participants. By employing multivariable logistic regression, subgroup analysis, and restricted cubic spline plots, a significant inverse association was found between GNRI and the prevalence of depression in older adults. A higher GNRI was established as a key protective factor against the development of depression in this population and was also shown to be a predictive indicator of depression risk in elderly individuals.

## Data Availability

The datasets utilized in this study are acquired from the publicly accessible NHANES database. (https://www.cdc.gov/nchs/nhanes/)

## Abbreviations

GNRI: Geriatric Nutritional Risk Index
NHANES: National Health and Nutrition Examination Survey
CDCP: Centers for Disease Control and Prevention
NCHS: National Center for Health Statistics
GNRI: Geriatric Nutritional Risk Index
DSM-IV: Diagnostic and Statistical Manual of Mental Disorders, Fourth Edition
PHQ-9: 9-item Patient Health Questionnaire
CVD: Cardiovascular Disease
CHF: Congestive Heart Failure
CHD: Coronary Heart Disease
SBP: Systolic Blood Pressure
DBP: Diastolic Blood Pressure
HDL: High-Density Lipoprotein
LDL: Low-Density Lipoprotein
MEC: Mobile Examination Center
PIR: Poverty Income Ratio
BMI: Body Mass Index
CVD: Cardiovascular Disease
OR: Odds Ratio
